# Analgesic, Antioxidant, Anti-Inflammatory, and Wound-Treating Actions of Bitter Apricot Kernel Extract

**DOI:** 10.1155/2024/5574259

**Published:** 2024-08-31

**Authors:** Mohamed Amine El-Hajjaji, Kawtar Fikri-Benbrahim, Najoua Soulo, Ghizlane Nouioura, Hassan Laaroussi, Pedro Ferreira-Santos, Badiaa Lyoussi, Zineb Benziane Ouaritini

**Affiliations:** ^1^ Laboratory of Natural Substances, Pharmacology, Environment, Modeling Health and Quality of Life Faculty of Sciences Sidi Mohamed Ben Abdellah University, Fez 30000, Morocco; ^2^ Laboratory of Microbial Biotechnology and Bioactive Molecules, Sciences and Technologies Faculty Sidi Mohamed Ben Abdellah University, P.O. Box 2202, Imouzzer Road, Fez, Morocco; ^3^ Department of Chemical Engineering Faculty of Science University of Vigo, As Lagoas, Ourense 32004, Spain; ^4^ IAA-Instituto de Agroecoloxía e Alimentación University of Vigo (Campus Auga), As Lagoas, Ourense 32004, Spain

## Abstract

Apricot (*Prunus armeniaca* L.) kernels have been widely employed in phytomedicine for treating different ailments. This study aims to unveil the phytochemical composition by HPLC-ESI-MS, *in vitro* antioxidant activity, and examine certain pharmacological effects of the hydro-ethanolic extract from bitter apricot kernels (BAK). Obtained results indicated that the BAK extract presents a content of 4.58 ± 0.15 mg GAE/g extract of TPA and 1.68 ± 0.09 mg QUE/g extract of TFA, respectively. HPLC-ESI-MS analysis discovered the presence of 17 phenolic compounds including phenolic acids and flavonoids like 3,4-dihydroxybenzoic acid, gallic acid, caffeic acid, (+)-catechin, epicatechin, and others, with associated antioxidant power. Regarding the studied potential pharmacological effects, notable analgesic activity at a dosage of 100 mg/kg BW was recorded with 63.46% protection. In the anti-inflammatory test, significant inhibition was observed after 6 hours of treatment (77.4%) compared to untreated animals. Moreover, the daily application of ointment formulated with 10% BAK extract resulted in a remarkable healing of wounds and burns in rats. These findings underscore the increasing evidence supporting the potential use of apricot kernel extracts in treating various diseases.

## 1. Introduction

Plants have been utilized historically as medicinal remedies for a range of ailments and health disorders. As a potent remedy for numerous ailments, medicinal plants and their by-products continue to hold a significant position in modern medicine [1]. In recent years, consumers have increasingly expressed concerns regarding the inclusion of synthetic additives, namely, antioxidants butylated hydroxytoluene (BHT) and butylated hydroxy-anisole (BHA) in food. Research has indicated that these commonly used antioxidants can induce DNA damage [2]. Consequently, there has been a significant focus on exploring natural sources of effective antioxidant compounds. In this line, recently, extensive efforts have been made to identify compounds with potential antioxidant properties that could serve as suitable replacements for synthetic additives in food, cosmetic, and pharmaceutical industries. Plant species are rich in bio-functional components, such as vitamins, flavonoids, carotenoids, and phenolic acids. These elements, commonly found in the human diet, have shown robust antioxidant activities [3].

Plants have long been recognized for their potential to alleviate pain and discomfort through their analgesic properties. A wide range of plant species have been studied for their analgesic properties because of their diverse phytochemical substance, which includes phenolics, alkaloids, and terpenoids [4, 5]. The analgesic efficacy of these plant-derived compounds has been demonstrated in various preclinical and clinical studies [6]. These bio-based substances exert their analgesic effects through diverse mechanisms, including modulation of neurotransmitter pathways, anti-inflammatory actions, and inhibition of pain mediators. The exploration of plant-based analgesics continues to be an active area of research, with the aim of developing safer and more effective pain management strategies that harness the therapeutic potential of nature's pharmacopeia [7, 8].

Inflammation is a physiological response of tissue blood vessels to an inciting agent, marked by the influx of fluids and cells into the interstitial spaces. This inflammatory process is distinguished by cardinal signs, including heat, redness, pain, and loss of function [9]. While the causes of inflammation may vary, the underlying mechanisms remain consistent across diverse etiologies. Numerous studies have substantiated that in various regions across the world, plants are extensively employed in traditional medicine to alleviate various inflammatory disorders [10–14].

Burns continue to be regarded as one of the most challenging medical conditions, impacting individuals across all age groups in both developed and developing countries [15]. Wound treatment is a complex and intricate biological process that comprises four essential steps: homeostasis, proliferation, inflammation, and maturation. Each phase is important for the complete healing and regeneration of injured tissue, contributing to the successful healing of wounds [16]. Several factors can contribute to the delay in the wound repair process, encompassing the size and severity of the wound, the individual's overall health status, and the presence of underlying medical conditions. Additionally, oxidative stress assumes a significant role in impeding healing and exacerbating tissue damage [17].

The apricot species (*Prunus armeniaca* L.) of the Rosaceae family is not widely distributed because it can only grow in selected areas with ideal environmental conditions. It is native to China and Japan, although it is grown in Morocco and other temperate to subtropical regions around the world [18]. Apricot kernels have earned considerable acclaim for their therapeutic potential, and these seeds have been utilized in the treatment of various diseases, including bronchitis, asthma, emphysema, constipation, nausea, leprosy, leukoderma, and pain [19]. Furthermore, bitter apricot kernels (BAK) have also demonstrated efficacy in addressing diverse skin conditions [20] and exhibited several pharmacological effects, such as antimicrobial, antioxidant, antiasthmatic, anticancer, antianalgesic, and anti-inflammatory activities [21, 22].

Ostensibly, no published investigations examine the phytochemicals and pharmacological activities of Moroccan cultivar apricot kernels. The current study aims to assess the phytochemical profile, anti-inflammatory, analgesic, antioxidant, and wound treatment properties of the BAK hydro-ethanolic extract, aiming to enhance our understanding of its potential therapeutic benefits and explore its applications across diverse domains.

## 2. Materials and Method

### 2.1. Reagents

2,2-Diphenylpicrylhydrazyl (DPPH), aluminum chloride, ethanol, Folin–Ciocalteu, quercetin, ascorbic acid, sodium bicarbonate, sodium hydroxide, phosphate buffer, sodium nitrite, potassium ferricyanide, trichloroacetic acid, acetic acid, ferric chloride, and gallic acid were purchased from Sigma-Aldrich (Munich, Germany).

### 2.2. Plant Material

Apricot fruits (*Prunus armeniaca* L.) of the Maoui variety were obtained from the Sefrou region Morocco (33°40′13.6″N 4°51′28.7″W) in June 2022. The studied plant was recognized by Pr. Amina Bari, a Botanist from Sidi Mohamed Ben Abdellah University (USMBA), and the voucher specimen was under the following code: RPA 001 VM 226 SL. The stones were removed from the fruits, and the kernels were extracted and dried for one week at ambient temperature. Finally, the kernels were stranded into a fine powder utilizing an electric mixer and the powder was kept at 4°C until used.

### 2.3. Extract Preparation

10 g of BAK powder was saturated in 100 mL of 70% ethanol-water (v/v) for 48 h with continuous agitation at 200 rpm. After filtration with Whatman filter paper (no. 1), the obtained filtrate was dried (yield: 6.25% of BAK), and the resultant extract was kept at 4°C for further experiments [23].

### 2.4. Identification of Phytochemicals

#### 2.4.1. Determination of Total Phenolic Amount (TPA)

The method outlined by Amin et al. was used to calculate the TPA of the examined extract [24]. For the experiment, 500 *μ*L of Folin–Ciocalteu reagent was mixed with 100 *μ*L of the diluted sample, and then, 400 *μ*L of sodium carbonate (7.5% w/v) was added to the obtained mixture. The absorbance was measured at 760 nm after two hours of incubation in the dark at room temperature. TPA was estimated by making a calibration curve with gallic acid (*y* = 3.0533*x* + 0.045; *R*^2^ = 0.99), and it was calculated as milligrams of gallic acid equivalent per gram of extract (mg GAE/g extract).

#### 2.4.2. Determination of Total Flavonoid Amount (TFA)

The TFA was determined using the aluminum chloride colorimetric method previously described by Bahorun et al. [25] with some changes. To test, 1.5 mL of the BAK extract was mixed with 1.5 mL of 2% AlCl_3_. Following a 30-minute incubation in the dark, the absorbance was measured at 430 nm. The TFA was calculated with a calibration curve prepared with quercetin (*y* = 41.129*x* + 0.1506; *R*^2^ = 0.99) and expressed as milligrams of quercetin equivalent per gram of extract (mg QUE/g extract).

#### 2.4.3. Identification of Phenolic Compounds by HPLC-ESI-MS

Phenolic constituents were pointed out by high-performance liquid chromatography/electrospray ionization/mass spectrometry (HPLC/ESI/MS) using an HPLC (Agilent 1260 series) with AB SCIEX Triple Quad 3500 detector (AB Sciex) associated with an electrospray ionization source. A volume of 5 *μ*L was injected using two mobile states (0.1% formic acid without and with acetonitrile) at a flow level of 0.3 mL/min, employing a Luna C18 column (Phenomenex). Ionization was carried out using a negative-positive source with Turbo V™, and N served as both the collision and nebulizer gas. Multiple reaction monitoring (MRM) was employed to collect data. Results were expressed in micrograms of each compound per gram of BAK extract (*μ*g/g).

### 2.5. Antioxidant Activity

#### 2.5.1. 2,2-Diphenyl-1-picrylhydrazyl Radical Assay (DPPH)

The antioxidant effect of the hydro-ethanolic extract was measured with the free radical scavenging method using stable 2,2′-diphenyl-1-picrylhydrazyl radical solution (DPPH at 150 *μ*M) and prepared in ethanol with an absorbance of 0.700 ± 0.01 at 517 nm [26]. To begin the test, a sequence of dilutions (2-fold) was created across 10 test tubes. Subsequently, 50 *μ*L of diverse concentrations of the BAK extract was introduced into 825 *μ*L of the DPPH solution. After 20 minutes without light at room temperature, the absorbance was measured at 517 nm. Ascorbic acid served as the standard solution. Equation (1) was used to determine the percentage of DPPH radical inhibition. The DPPH inhibition concentration at 50% (IC_50_) was determined by examining the graph indicating the extract's inhibition percentage and expressed in milligrams of extract per mL (mg/mL):

(1) $$\begin{array}{c} \text{inhibition}\left(\%\right)=\frac{\text{Abc}-\text{Abs}}{\text{Abc}}\times 100, \end{array}$$

where Abc represents the absorbance of the control and Abs represents the absorbance of the BAK extract or ascorbic acid.

#### 2.5.2. Antioxidant Reducing Power Assay (RP)

The ferric reducing antioxidant power of the BAK extract was estimated following the protocol of Oyaizu [27]. Briefly, a sample of 0.2 mL was mixed with 0.5 mL of phosphate buffer solution (0.2 M, pH 6.6) and 0.5 mL of potassium ferricyanide (1%) (w/v). After 20 minutes in a 50°C water bath, 0.5 mL of trichloroacetic acid (10%) (w/v) was supplemented, and the formulation was centrifuged at 3000 rpm for 10 minutes. Next, 0.5 mL of the supernatant was mixed with 0.5 mL of water distillated and 0.1 mL of a FeCl_3_ solution (0.1%) (w/v), and the absorbance of the mixture was determined at 700 nm. The resulting data were expressed as effective concentrations. From the graph, the median effective concentration (EC_50_) was calculated and expressed as milligrams of extract per mL (mg/mL).

### 2.6. Pharmacological Activities

#### 2.6.1. Experimental Animal's Protocol

Male and female Swiss mice (25–30 g) and Wistar rats (200–230 g) were provided from the Animal Breeding Center of the Faculty of Sciences Dhar El-Mahraz, USMBA, Fez, Morocco. Animals in this study were housed in a controlled setting with a temperature of 26 ± 2°C and a 12-hour photoperiod (12 L/12 D). They had unrestricted access to tap water and food throughout the duration of the experiment. The ethical protocols followed in this study were approved by our institution's animal protection committee, in accordance with the guidelines outlined in the ethical approval registration under the number L.20. USMBA-SNAMOPEQ 2020-03.

#### 2.6.2. Analgesic Activity

To assess potential peripheral effects stemming from the hydro-ethanolic of BAK extract, the acetic acid-induced writhing test was implemented in accordance with the methodology detailed by Okun and Liddon [28]. A total of fifteen mice, comprising both sexes and weighing between 20 and 25 g, were utilized for the study and systematically divided into three groups, each consisting of five mice (*n* = 5):

  Group 1: normal saline solution, 0.9% NaCl (control)
  Group 2: diclofenac, 25 mg/kg of body weight (b.wt) (analgesic standard)
  Group 3: hydro-ethanolic BAK extract, 100 mg/kg b.wt

Thirty minutes after the administration of tested substances, each mouse received an intraperitoneal injection of 0.1 mL/10 g b.wt of a 0.7% acetic acid solution. Subsequently, the mice were positioned within transparent observation boxes for the purpose of scrutinizing writhing responses, encompassing manifestations such as contraction of abdominal musculature, trunk twisting, back arching, hind limb extension, and torsion to one side resulting in the mouse's belly contacting the floor, during 30 minutes. All mice's writhes were counted within a period of thirty minutes, and the formula below (2) was utilized to calculate the protection percentage (%) against writhing.

(2) $$\begin{array}{c} \text{PP}\left(\%\right)=\frac{\text{Mc}-\text{Mt}}{\text{Mc}}\times 100, \end{array}$$

where Mc represents the abdominal contraction's mean number in the control group and Mt represents the standard group or BAK extract-treated group.

#### 2.6.3. Anti-Inflammatory Activity

BAK extract was tested for anti-inflammatory effects following the procedure outlined previously [29]. Fifteen Wistar rats weighing between 200 and 230 g were used for the study and separated into three equal groups, each group consisting of five rats (*n* = 5):

  Group 1: normal saline solution, 0.9% NaCl (control)
  Group 2: indomethacin 10 mg/kg b.wt (standard)
  Group 3: hydro-ethanolic BAK extract (100 mg/kg)

Thirty minutes after the administration of the tested compounds, all rats received a subcutaneous injection of carrageenan (0.5%) in their right paw, with a volume of 0.1 mL to induce edema. A digital Vernier caliper (Moge, PRC) was used to measure each rat's paw thickness (in millimeters) at 2, 3, 4, 5, and 6 hours. The paw thickness measurement is commonly used as a reliable indicator of inflammation in experimental studies [30]. The percentage of inflammation inhibition (PI) was calculated as follows:

(3) $$\begin{array}{c} \text{PI}\left(\%\right)=\frac{\left(\text{Dt }-D0\right)\text{ Control}-\left(\text{Dt }-\;D0\right)\text{ treated}}{\left(\text{Dt }-D0\right)\text{ Control}}\times 100. \end{array}$$

The mean diameter of the paw before injection was represented by D0 and after the injection of carrageenan at a given time by Dt.

#### 2.6.4. Wound Healing

*(1) Preparation of Ointment*. The process elucidated by Mssillou et al. [31] was followed in order to prepare the ointment. Nine grams of Vaseline® (Unilever, UAE) was mixed with one gram of hydro-ethanolic extract of BAK. The mixtures were transferred to glass beakers placed in a water bath at 50°C and were stirred continuously until thoroughly mixed. Up to use, the ointment was kept at 4°C.

*(2) Wound Healing Activity*. Wound healing activity of the studied extract was evaluated on both male and female Wister rats following the protocol as described by Mssillou et al. [31]. Briefly, the rats were separated into three groups, each group contained three rats, and then, the animal's dorsum hair was shaved and anesthetized by intraperitoneal injection with Pentobarbital (50 mg/kg b.wt). In order to create a deep second-degree burn, a metal cylinder with 3 cm in diameter and heated at 100°C was used. The ointments were applied daily to the whole wound surface for 21 days starting 24 hours after the burns were performed.

  Group 1: Vaseline® (control)
  Group 2: Biaffine® 1% (standard)
  Group 3: Ointment formulated with 10% of BAK hydro-ethanolic extract

The results were taken daily for three weeks. All rats were kept under standard laboratory conditions [32] and obtained the same food and drink during the study period. ImageJ software was used to analyze the obtained images, and equation (4) was utilized to measure the rate of contraction of the wound surfaces (Wc).

(4) $$\begin{array}{c} \text{Wc}\left(\%\right)=\frac{\left(\text{Ws}0-\text{Ws}1\right)\;}{\text{Ws}0}\times 100. \end{array}$$

The wound's size on the first day was represented by Ws0 and on each specific day by Ws1.

### 2.7. Statistical Analysis

Statistical analysis, including Dunnett's multiple comparisons and one-way ANOVA tests, was performed by GraphPad Software Inc. (version 9.2.0.332, San Diego, CA, USA). Results were presented as a mean standard deviation.

## 3. Results and Discussion

### 3.1. Characterization of BAK Extract

Polyphenols, natural compounds found in plants, are renowned for their various properties, such as antibacterial, anti-inflammatory, and antioxidant activities. Apricots have been discovered to contain substantial levels of polyphenols, as well as their subclass, flavonoids [33]. The results obtained for TPA and TFA of the BAK hydro-ethanolic extract were 4.58 ± 0.15 mg GAE/g extract and 1.68 ± 0.09 mg QUE/g extract, respectively. The obtained results are higher than those carried out by Gomaa [34] who found that the TPA and TFA of the ethanolic extract of sweet apricot kernels were to be 1.004 ± 0.6 mg GAE/g extract and 0.468 ± 0.1 mg QUE/g extract, respectively. Moreover, Yigit et al. observed that the TPA in the water extract of bitter apricot kernels was measured at 0.4 ± 0.1 *μ*g GAE/mL [35]. Conversely, a notably higher TPA value of 7.9 ± 0.2 *μ*g GAE/mL was documented for the sweet apricot kernels. In addition, the study carried out by Sochor et al. [36] on 21 new apricot genotypes revealed that the total polyphenol levels were varied from 41 to 170 mg GAE/100 g FW. TPA and total antioxidant capacity have previously been shown to be affected by genotype variation in fresh apricot fruit [37], almond [38], hybrid berry, and blackberry [39].

The BAK hydro-ethanolic extract was subjected to HPLC-ESI-MS analysis to determine its phenolic compound composition.  Table 1 provides a comprehensive summary of the identified phenolic compounds, detailing their retention times, molecular formulas, and name of tentatively identified compounds. Sixteen phenolic compounds were identified, encompassing phenolic acids and flavonoids, among others. Phenolic acids are found in diverse plant tissues, such as fruits, leaves, roots, and vegetables [40]. This study identified several phenolic acids, including 3,4-dihydroxybenzoic acid, gallic acid, syringic acid, and vanillic acid, in the provided extract. Among these, 3,4-dihydroxybenzoic acid was found to be the predominant phenolic acid, with a concentration of 345.18 ± 3.19 *μ*g/g extract. Additionally, flavonoid compounds such as naringenin, catechin, luteolin, and epicatechin were detected and quantified in the extract, with catechin being the most abundant flavonoid component at 153.10 ± 0.82 *μ*g/g extract. These compounds have been largely reported in the literature for their antioxidant capacity [41]. Furthermore, hydroxycinnamic acids, such as caffeic acid, ferulic acid, and *p*-coumaric acid commonly found in numerous medicinal plants and functional foods, have been documented to offer expansive health benefits, notably anti-inflammatory and antiviral effects [42]. Catechin is a bio-valuable phytochemical that has shown promising results in enhancing overall health and treating a variety of ailments and health disorders. It can modulate the Nrf2 and NFk*β* pathways, which explain its antioxidative effects and anti-inflammatory properties [43]. It was reported that protocatechuic acid (3,4-dihydroxybenzoic acid) effectively reduced nitric oxide production in microglial cells stimulated with lipopolysaccharide, indicating its effectiveness as an anti-inflammatory agent [44]. Caffeic acid stands as the predominant phenolic acid prevalent in fruits, whereas ferulic acid exists in esterified form within the cell walls of the seed coat, bran, and fruits [45]. *p*-Coumaric acid, also known as 4-hydroxycinnamic acid, has shown promising pharmacological effects, encompassing antiproliferative, nephroprotective, neuroprotective, antioxidant, and antimicrobial properties, alongside various other biological attributes [46]. Moreover, rutin, also known as quercetin-3-rutinoside, is widely contained in various plants, such as passion flowers, buckwheat, and apples. It has been extensively investigated for its pharmacological properties, including analgesic, anticancer, anti-inflammatory effects, and wound healing activity [47]. The phenolic profile of wild apricot kernel skins was previously investigated by Qin et al. [48], who identified approximately 35 polyphenol compounds, comprising phenolic acids, flavonoids, and anthocyanins. Salicylic acid and gentisic acid were the most quantitatively substantial among the total phenolic acids, measuring at 31.34 and 38.92 mg/100 g, respectively. Prior research investigated by Mandalari et al. [49] reported that catechin, epicatechin, kaempferol-3-*O*-rutinoside, quercetin-3-*O*-rutinoside, and kaempferol-3-*O*-glucoside were the major flavonoids in almond skins. Furthermore, hydroxybenzoic acids including vanillic, chlorogenic, *p*-hydroxybenzoic, and trans-*p*-coumaric acids were identified. In addition, the study performed by Al Juhaimi et al. [50] revealed that gallic acid, (+)-catechin, 3,4-dihydroxybenzoic acid, apigenin-7-glucoside, syringe, caffeic acid, 1,2-dihydroxybenzene, rutin, and quercetin were the main phenolics detected in wild apricot kernel samples, and some of these phenolic compounds were reduced a cause to the roasting process in microwave at 720 W.

### 3.2. Antioxidant Activity

**Figure 1:**
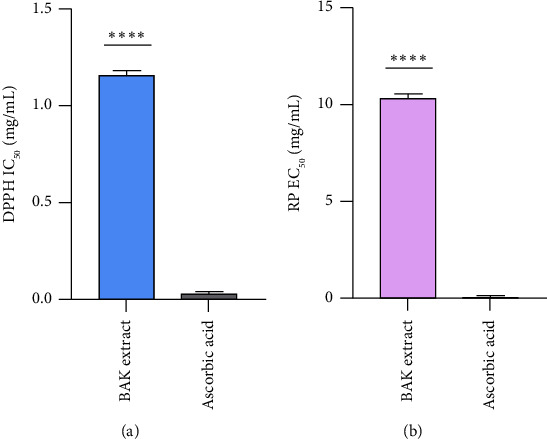
Antioxidant activity of bitter apricot kernel extract using DPPH (a) and RP (b) assays.

The antioxidant activity of the selected extract was evaluated using the DPPH scavenging capacity and the RP assays, and they were expressed as IC_50_ and EC_50_, respectively. The studied BAK extract showed a moderate effect (Figure 1), as it was active against the DPPH radical with an IC_50_ value of 1.17 ± 0.06 mg/mL (A). Indeed, this antioxidant activity of the studied extract is lower than that of ascorbic acid (IC_50_ = 0.043 ± 0.001 mg/mL) (*P* ≤ 0.0001), since lower IC_50_ value indicates a more potent antioxidant effect. Furthermore, the RP assay findings (B) demonstrated that hydro-ethanolic BAK extract has a lower potential to reduce iron (EC_50_ = 10.44 ± 0.08 mg/mL) when compared to the ascorbic acid (EC_50_ = 0.08 ± 0.01 mg/mL) (*P* ≤ 0.0001). Earlier work realized by Qin et al. [48] highlighted the antioxidant effect of apricot skin extracts. These extracts exhibited significant results of DPPH free radical scavenging capacity (IC_50_ = 13.77 ± 0.98 *μ*g/mL), reducing activity (EC_50_ = 3.05 ± 0.78 *μ*g/mL), and ABTS scavenging capacity (IC_50_ = 0.24 ± 0.23 *μ*g/mL). Furthermore, Gomaa's findings revealed that the ethanolic extract of sweet apricots and bitter almond kernels displayed remarkable levels of radical scavenging activity, with values of 51% and 62%, respectively [34]. The remarkable antioxidant capacity of BAK extract can be credited to the chemical features of the polyphenols. These encompass multiple hydroxyl groups, their specific arrangement, and the inclusion of supplementary substituents like double bonds, glycosylation, and conjugation. These elements function as reducing agents, singlet oxygen quenchers, hydrogen donors, and scavengers of free radicals [51]. Other works revealed a significant link between TPA and antioxidant property in apricots, peaches, nectarines, and plum fruits [52, 53]. Furthermore, various phenolic acids and flavonoids from the apricot kernel extracts, such as caffeic, gallic, gentisic, and salicylic acids, along with quercitrin and kaempferol, have demonstrated potent antioxidant activity against DPPH, ABTS, and superoxide radicals [48].

### 3.3. Pharmacological Activities

#### 3.3.1. Analgesic Activity

Using the acetic acid-induced writhing procedure on mice, the hydro-ethanolic extract of BAK was tested for its peripheral antinociceptive activity. According to the results in Table 2, oral use of the tested BAK extract at 100 mg/kg b.wt showed a significant analgesic action (*P* ≤ 0.001) with a protection percentage of 63.46% in contrast to the control group (group 2). A protection percentage of 93.30% was observed for the group administered with diclofenac sodium at an amount of 25 mg/kg b.wt against writhing produced by glacial acetic acid. The findings are consistent with those disclosed previously by Badr et al. [54] who mentioned that apricot kernel extract had a significant analgesic activity on tested rats when administered orally. Furthermore, the outcomes obtained by Ramadan et al. [55] demonstrated that ethanolic extracts given orally (at 100 mg/kg b.wt) exhibited a notable analgesic effect, providing 68.99% protection against writhing induced by glacial acetic acid. Moreover, there were no instances of mortality or toxic symptoms observed in animals orally administered the ethanolic extract of apricot kernels, and the LD_50_ was determined to be higher than 10 g/kg b.wt. The mechanism underlying the analgesic effect appears to be rooted in the inhibition of endogenous substance release, specifically those that activate pain nerve endings. This intricate process is likely mediated peripherally, suggesting that the alleviation of pain is intricately tied to the regulation of signaling molecules at the site of pain perception. By intervening in the periphery, this proposed mechanism aims to finely tune the modulation of pain-inducing substances, offering a potential avenue for targeted therapeutic interventions focused on mitigating pain at its source [56].

#### 3.3.2. Anti-Inflammatory Activity

**Figure 2:**
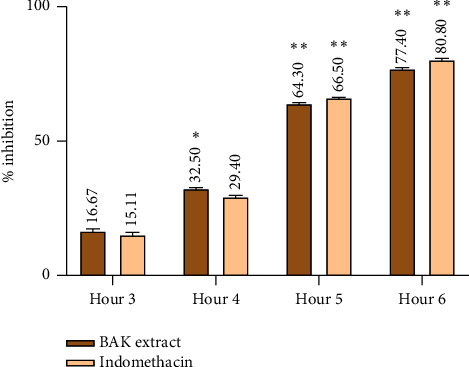
Anti-inflammatory activity of BAK extract and indomethacin. Significant results are represented at ^∗^*P* ≤ 0.05 and ^∗∗^*P* ≤ 0.005.

Through intraperitoneal injection of carrageenan, rats' paw edema was employed to test the *in vivo* anti-inflammatory property of the studied BAK extract.  Figure 2 illustrates the changes in edema observed in the rats' hind paws in millimeters after administration of physiological water, standard anti-inflammatory medication (indomethacin), and the BAK extracts. All rats exhibited comparable levels of edema at the beginning of the experiment. After four hours, rats administered the hydro-ethanolic extract showed less thickness on their inflamed paws (*P* ≤ 0.05). After six hours, in comparison with the control group, both the hydro-ethanolic BAK extract and standard groups exhibited a significant reduction in the thickness of the inflamed rat paws with 77.4% and 80.8%, respectively. These results align with previous findings by Ramadan et al. [55], who demonstrated that the inflamed rat paw thickness was significantly decreased when the ethanolic extracts of apricot kernels were orally administrated. In addition, the current findings align with the earlier research conducted by Badr et al. [54], which demonstrated strong anti-inflammatory activity in rats through the use of the extract derived from apricot kernels in a histamine-induced paw edema model. Previous investigation of Kim et al. [57] on the therapeutic effect of the topical application of apricot kernel extract on dry eye found that the expression level of TNF-*α* in mice treated with apricot kernel extract was significantly reduced, and the inflammatory reaction in the mice with dry eye decreased as well. The research done by Esposito et al. [58] highlighted that the acute inflammatory response is characterized by edema resulting from enhanced vascular permeability, resulting in the leakage of fluid and infiltration of leukocytes. During the initial phase of inflammation, histamine, serotonin, and bradykinin play a role, while prostaglandins, primarily induced by cyclooxygenase (COX)-2 in the inflamed tissue, sustain the late phase of inflammation. The obtained anti-inflammatory property of the investigated extracts can be attributed to their potential to inhibit the synthesis of proinflammatory cytokines and COX-2, and to reduce prostaglandin synthesis [21].

#### 3.3.3. Wound Healing

**Figure 3:**
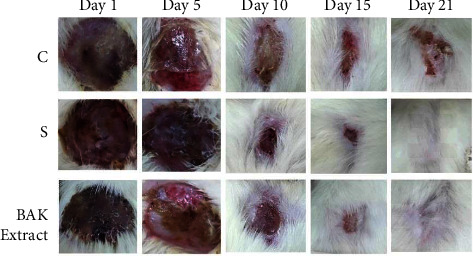
Pictures of animal wound areas on 1st, 5th, 10th, 15th, and 21st day of the experiment. C: control; S: standard.

**Figure 4:**
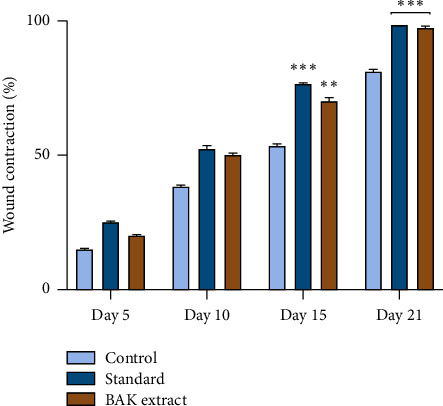
Wound contraction rate (%) in 5th, 10th, 15th, and 21st day of treatment. Significant values are represented at ^∗∗^*P* ≤ 0.01 and ^∗∗∗^*P* ≤ 0.001.

The wound-treating property of BAK extracts was tested through the experimental second-degree burns in Wistar rats. The obtained results during the 1st, 5th, 10th, 15th, and 21st days are presented in Figures 3 and 4. The topical application of the studied extract and the standard medication (Biaffine® 1%) on wounds demonstrated that the wound healing process progressed significantly faster than the control group (Vaseline®). Moreover, after one week of topical application of the prepared BAK extract ointment, all dead tissues were eliminated, and the lesion's size was reduced, and after three weeks of its topical application, significant healing activity was recorded, which induced complete tissue repair and wound closure (97 ± 0.9%). On the other hand, in the nontreated group (only Vaseline®), wounds in the rats did not heal totally at the end of treatment. Abdulsamad et al. [59] indicated that apricot seed extract showed excellent healing of eye corneal ulcers of pigeons. The important healing effect observed for the studied extract could be explained by the fact that the kernels of bitter apricots are rich in bioactive compounds, namely, alkaloids, flavonoids, phenolic acids, and tannins [60, 61]. Earlier findings demonstrated the beneficial effects of apricot kernels in the acceleration of the wound-treating process of burned skin in Wistar rats [62], and this study indicated the presence of signs of wound healing as neovascularization and the beginning of reepithelialization, as well as a complete epidermal epithelialization and increased fibrosis in the dermis of sections from the group treated with apricot kernels. Furthermore, numerous reports in the literature about the usage of medicinal plant extracts as an effective treatment to promote wound healing [31, 63–66]. Several physiological processes are involved in the healing effect, including hemostatic and inflammatory mechanisms, cell migration and proliferation, wound contraction, protein synthesis, and tissue remodeling or reconstitution. Moreover, many studies have shown the involvement of phytochemicals in these different mechanisms that contribute to the healing process [67, 68].

## 4. Conclusion

The discoveries unveiled by this investigation reinforce the belief in multifaceted medicinal properties harbored within apricot kernels, holding promise for human health improvement. Several bioactive polyphenols were identified in the BAK extract and exhibited remarkable antioxidant activity, notable potential in alleviating pain and inflammation, and demonstrating significant efficacy in treating burns and expediting the healing process. Delving deeper into the mechanisms underpinning these pharmacological effects could open doors to pioneering treatments leveraging the therapeutic potential of apricot seeds. However, to validate these findings, further exploration involving elevated doses, varied administration routes, and extended durations remains imperative. Moreover, a comprehensive assessment encompassing bioactive constituent's toxicity should be meticulously run. Furthermore, cautious consideration regarding potential impacts on blood parameters is paramount before contemplating the utilization of BAK extracts.

## Figures and Tables

**Table 1:** Identified compounds in hydro-ethanolic extracts of bitter apricot kernels.

Compounds	RT (min)	Molecular formula	Concentration (*μ*g/g extract)
Gallic acid	4.1	C_7_H_6_O_5_	13.09 ± 0.14
3,4-Dihydroxybenzoic acid	8.2	C_7_H_6_O_4_	345.18 ± 3.19
Theobromine	8.5	C_7_H_8_N_4_O_2_	9.62 ± 0.48
Theophylline	9.1	C_7_H_8_N_4_O_2_	0.06 ± 0.00
4-Hydroxybenzoic acid	9.6	C_7_H_6_O_3_	10.44 ± 0.66
Caffeic acid	9.9	C_9_H_8_O_4_	1.10 ± 0.08
Syringic acid	10.4	C_9_H_10_O_5_	6.06 ± 0.15
Rutin	10.7	C_27_H_30_O_16_	35.08 ± 2.10
*p*-Coumaric acid	11.0	C_9_H_8_O_3_	4.23 ± 0.13
Syringaldehyde	11.2	C_9_H_10_O_4_	88.73 ± 3.57
Ferulic acid	11.3	C_10_H_10_O_4_	1.84 ± 0.06
Catechin	11.6	C_15_H_14_O_6_	153.10 ± 0.82
Vanillic acid	12.1	C_8_H_8_O_4_	26.23 ± 0.72
Epicatechin	12.4	C_15_H_14_O_6_	1.31 ± 0.24
Luteolin	12.5	C_15_H_10_O_6_	14.65 ± 0.56
Naringenin	12.8	C_15_H_12_O_5_	17.10 ± 0.40

**Table 2:** The analgesic activity of hydro-ethanolic extract of bitter apricot kernels.

Treatment	Dose (mg/kg b.wt)	Number of writhes in 30 min^a^	Protection (%)
Normal saline solution (control)	0	10.4 ± 1.07	0
Diclofenac sodium	25	0.80 ± 0.45^∗∗∗^	92.3
BAK extract	100	3.80 ± 1.64^∗∗∗^	63.5

^a^Values reported are means ± standard deviations of five animals from each group. Significant values are defined at ^∗∗∗^*P* ≤ 0.001 compared to the control group.

## Data Availability

The data used to support the findings of this study are available from the corresponding author upon reasonable request.
